# A Technique to Salvage Big-Bubble Deep Anterior Lamellar Keratoplasty after Inadvertent Full-Thickness Trephination

**Published:** 2011-01

**Authors:** Siamak Zarei-Ghanavati, Mehran Zarei-Ghanavati

**Affiliations:** Eye Research Center and Department of Ophthalmology, Khatam-al-Anbia Eye Hospital Mashhad University of Medical Sciences, Mashhad, Iran

**Keywords:** Deep Anterior Lamellar Keratoplasty, DALK, Big-Bubble, Full-Thickness Trephination

## Abstract

Herein we describe a technique for management of large inadvertent full-thickness trephination during deep anterior lamellar keratoplasty using the big-bubble technique without converting to penetrating keratoplasty. First, the anterior chamber is formed with an ophthalmic viscosurgical device (OVD). Then, the full-thickness wound is secured with one X-type 10-0 nylon suture. A 27-gauge needle is attached to a 2 ml air-filled syringe and inserted into the corneal stroma in the meridian opposite to the site of full-thickness trephination. Air is gently injected to produce a limited area of “big-bubble” detaching Descemet’s membrane (DM) from the corneal stroma. The “big bubble” is slowly expanded with injection of OVD. Finally, the recipient stroma is removed, the donor lenticule is placed and the DM tear is secured with one full thickness 10-0 nylon suture.

## INTRODUCTION

Deep anterior lamellar keratoplasty (DALK) using the big-bubble technique, in which dissection of corneal stroma down to Descemet’s membrane (DM) is achieved by air injection, was first described by Anwar and Teichmann^1^.

DM perforation is one of the common complications of DALK reported in 0%–15% of all cases^2^,^3^ and its occurrence is correlated with surgical experience. Large DM perforations due to inadvertent full-thickness trephination usually necessitate converting the procedure to penetrating keratoplasty (PK).

Herein, we describe a technique used in two patients to salvage deep anterior lamellar keratoplasty after large DM perforations during trephination.

## SURGICAL TECHNIQUE

**Figure 1:**
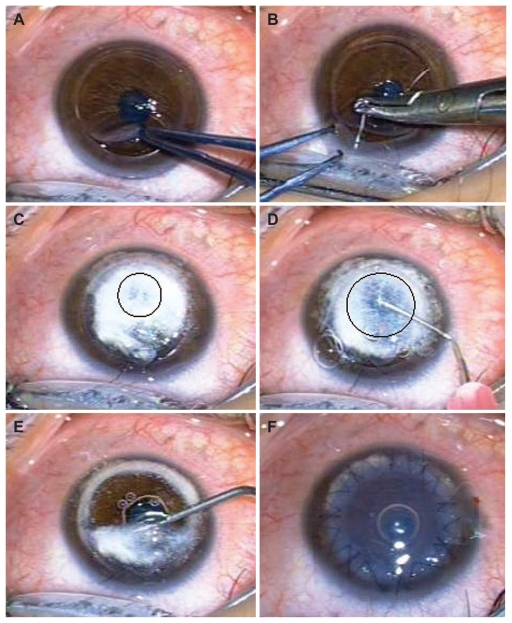
**(A)** Inadvertent full-thickness trephination from 11 to 1 o’clock with anterior chamber collapse. **(B)** The full-thickness wound is closed with one partial-thickness X-type 10-0 nylon suture. **(C)** A limited “big bubble” is formed after gentle air injection. **(D)** “Big bubble” expansion by OVD injection. **(E)** Release of remaining DM attachments by OVD injection. **(F)** The conclusion of surgery after securing the donor lenticule.

When we noted inadvertent full-thickness perforation (Fig. 1A) during trephination of the recipient cornea using a Hessburg-Barron suction trephine, the procedure was continued by forming the anterior chamber (AC) with an ophthalmic viscosurgical device (OVD). The full-thickness wound was secured with one partial-thickness X-type 10-0 nylon suture (Fig. 1B).

A bent 27-gauge needle was attached to a 2 ml air-filled syringe and inserted into mid-corneal stroma at the meridian opposite to the site of full-thickness perforation. Thereafter air was injected to form a limited area of “big bubble” detaching DM from the stroma (Fig. 1C). This “big bubble” was not expanded by air to avoid bubble collapse through the site of DM perforation. The needle was removed from the cornea and attached to an OVD syringe. Then, the needle was re-introduced into the bubble and OVD was injected progressively to separate DM from the stroma (Fig. 1D).

The residual stroma (bubble roof) was then punctured with a 15-degree blade and the stroma was cut into half using scissors. After removal of the bubble roof, any remaining attached DM was gently released by OVD injection into the pocket created between DM and stroma (Fig. 1E). Then the X-type nylon suture and residual stroma was removed. OVD was thoroughly washed from the DM and the donor lenticule was secured with 8 interrupted and one continuous 10-0 nylon sutures. One full-thickness suture was placed at the site of DM perforation to ensure attachment of DM to the stroma (Fig. 1F). The AC was completely filled with air through a limbal paracentesis for 5 minutes. At the end of surgery, half of the air was replaced by balanced salt solution.

## DISCUSSION

DM perforation is a common drawback of DALK. Full-thickness trephination is one cause of this complication, however its exact incidence is not clear. When full-thickness trephination occurs, the operation can be postponed; alternatively manual layer-by-layer dissection of the stroma can be performed in a pre-descemet plane^4^, or the procedure can be converted to PK as reported by Fontana et al^5^ in 4% of cases. Using our technique, after full-thickness wound closure with an X-type suture, a limited area of “big-bubble” is created by gentle air injection opposite to the site of DM tear in order to prevent expansion of the tear and bubble collapse when air reaches the perforation.

Den and colleagues^6^ reported double anterior chamber formation in all cases with DM macroperforations (larger than 1 mm). They injected air into the AC to impede pseudochamber formation. Shimmura^7^ suggested suturing DM to the donor graft at the site of a large perforation. We used one full-thickness suture at the site of DM tear to prevent double chamber formation.

One concern with large DM perforations is endothelial cell loss. Cell loss exceeding 20% was reported in a case of large DM tear leading to AC collapse.^8^ In both of our cases, OVD was injected into the anterior chamber to avoid further endothelial damage. Fortunately, at final follow-up, visual acuity was 20/40 in the first (Fig. 1) and 20/30 in the second case, and endothelial cell counts were 2961 and 2994 cells/mm^2^, respectively.

## Figures and Tables

**Figure 2:**
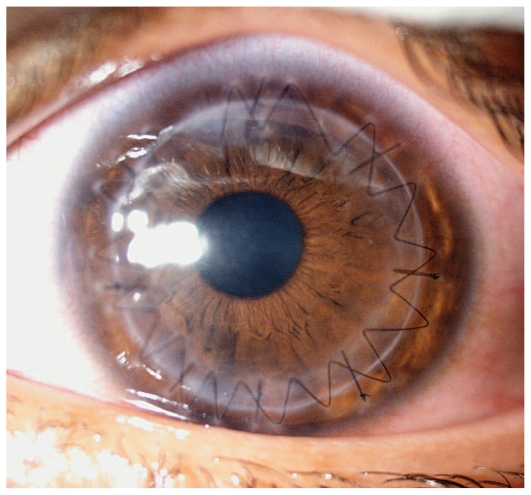
Same eye as in figure 1, two months after surgery.
